# Transient maintenance of tracheal patency upon the insertion of a flexible bronchoscope in a patient with an anterior mediastinal mass: a case report

**DOI:** 10.1186/s40981-021-00442-y

**Published:** 2021-04-29

**Authors:** Takayuki Hasegawa, Shinju Obara, Rieko Oishi, Satsuki Shirota, Jun Honda, Shin Kurosawa

**Affiliations:** 1 Department of Anesthesiology, Fukushima Medical University Hospital, 1 Hikarigaoka, Fukushima, Fukushima 960-1295 Japan; 2 Surgical Operation Department, Fukushima Medical University Hospital, 1 Hikarigaoka, Fukushima, Fukushima 960-1295 Japan

**Keywords:** Mediastinal mass, Airway obstruction, Flexible bronchoscopy, Veno-venous extracorporeal membrane oxygenation

## Abstract

**Background:**

Patients with an anterior mediastinal mass are at risk of perioperative respiratory collapse.

**Case presentation:**

A 74-year-old woman with a large anterior mediastinal mass that led to partial tracheal collapse (shortest diameter, 1.3 mm) was scheduled for tracheobronchial balloon dilation and stent placement under general anesthesia. Although veno-venous extracorporeal membrane oxygenation (V-V ECMO) had been established, maximum flow was limited to 1.6 L/min, and general anesthesia induction was followed by hypoxia probably due to inadequate ventilation. A flexible bronchoscope was inserted through the tracheal lumen that was being compressed by the anterior mass; this not only increased tracheal patency but also enabled positive pressure ventilation and resulted in recovery from hypoxia. Scheduled procedures were successfully performed without complications.

**Conclusion:**

We describe a case wherein tracheal patency was transiently maintained by inserting a flexible bronchoscope in a patient with an anterior mediastinal mass.

## Background

Patients with an anterior mediastinal mass are at risk of perioperative respiratory collapse. Induction of general anesthesia in such patients carries a particularly high risk because it not only affects airways and breathing but can also disrupt the patient’s physiological compensatory function [1]. Furthermore, in the absence of established consensus on general anesthesia management in such cases, regional anesthesia, rather than general anesthesia, may be indicated when possible, besides cooperation with an interdisciplinary team to secure cardiopulmonary bypass access and provide alternative airway and circulation management options [2].

Here, we describe a case where tracheal patency was transiently maintained by inserting a flexible bronchoscope in a patient with a narrowed trachea due to the presence of an anterior mediastinal mass. Patency was needed after the induction of general anesthesia and despite the establishment of veno-venous extracorporeal membrane oxygenation (V-V ECMO).

## Case presentation

**Fig. 1:**
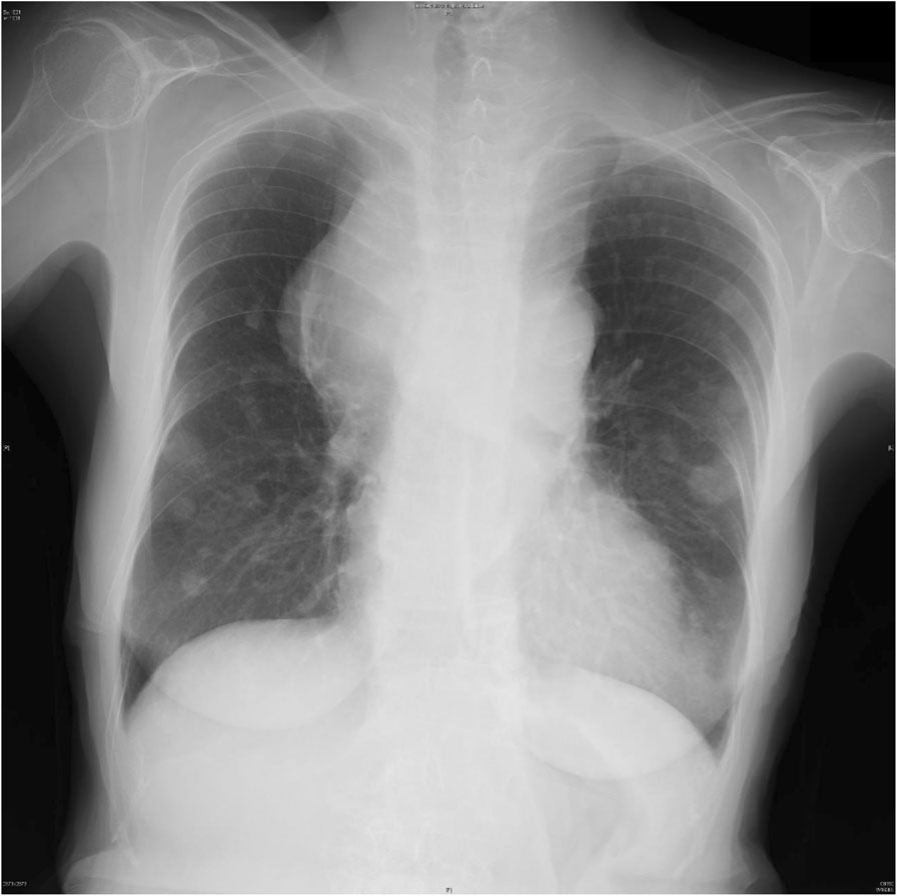
Chest X-ray before the procedure

**Fig. 2:**
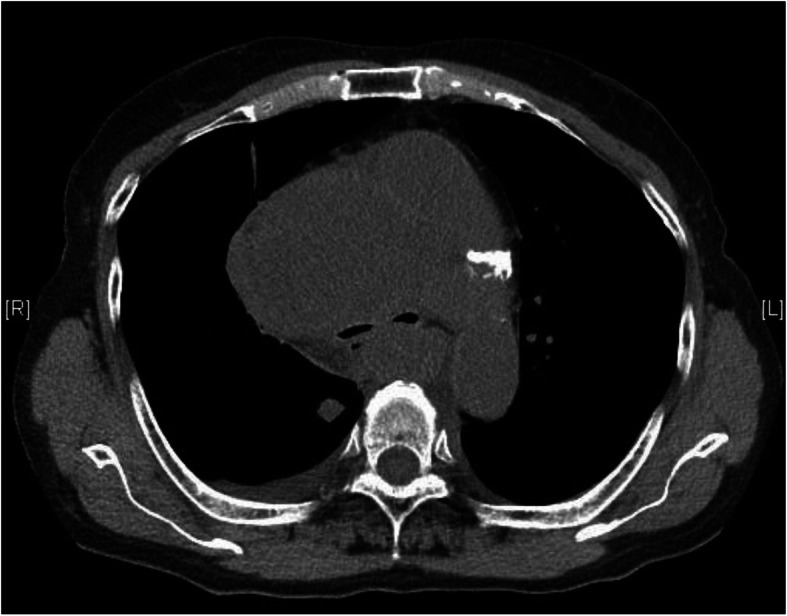
Preoperative chest computed tomography at the level of the main bronchus. Bilateral main bronchi were remarkably compressed by the anterior mediastinal tumor

Written informed consent was obtained from the patient for publication of this case report. A 74-year-old woman (body weight, 51 kg; height, 142 cm; and body surface area, 1.39 m^2^) with a large anterior mediastinal mass that was partially compressing her trachea was scheduled for emergent tracheobronchial balloon dilation and stent placement. She presented with 4 weeks of dyspnea, cough, and fatigue that had resulted in her being unable to maintain a supine position. She had been prescribed medication for hypothyroidism, which was under control, and had undergone resection of a uterine leiomyoma under spinal anesthesia, without any trouble, when she was 63 years old. Her dyspnea was alleviated in the left lateral decubitus position with a respiratory rate of 20/min and 97% pulse oxygen saturation (SpO_2_) on room air. She had a soft mass on the anterior portion of the neck, but there was no edema in the upper body. A chest X-ray revealed multiple bilateral pulmonary coin lesions and a mediastinal mass that had infiltrated the right upper lung field (Fig. 1). Preoperative chest computed tomography showed an anterior mediastinal mass, with a largest greatest dimension 90 mm, surrounding the main bronchus (Fig. 2) and the lower trachea, thereby compressing the trachea (shortest diameter, 1.3 mm). Both the superior vena cava and the inferior vena cava were compressed by the tumor. Laboratory tests revealed elevated lactate dehydrogenase (865 units/L), uric acid (9.4 mg/dL), blood urea nitrogen (37 mg/dL), and creatinine (2.3 mg/dL). Pulmonary function tests were not performed because of evident dyspnea. Preoperative bronchoscopy showed no intratracheal invasion of the tumor but the operator insisted on total immobilization using a muscular relaxant during the procedure because of safety concerns.

She was not administered with any anesthetic premedication and was placed in the left lateral decubitus position for surgery. The patient was monitored via continuous electrocardiography, pulse oximetry (SpO_2_), non-invasive blood pressure measurement, processed-electroencephalogram (SedLine®, Masimo, Irvine, CA, USA; version2000), and capnometry after tracheal intubation. For establishing V-V ECMO circulation, an infusion cannula (18 Fr) and a drainage cannula (20 Fr) were inserted into the right internal jugular vein and the right femoral vein, respectively under local anesthesia without sedation. V-V ECMO was initiated, but the maximum total flow rate reached was 1.6 L/min (31 mL/kg/min), probably because of limited drainage volume. After preoxygenation, general anesthesia was induced using propofol administered via Target Controlled Infusion (Diprifusor model, TE-371, Terumo, Tokyo, Japan) with a target plasma concentration of 3.0 μg/mL, along with remifentanil 0.25 μg/kg/min and rocuronium 50 mg. Inspiratory oxygen fraction (FiO2) was set at 1.0. The patient was shifted to the supine position, and mask ventilation was attempted but the tidal volume obtained was low at approximately 10 ml. Although tracheal intubation with an 8.0-mm internal diameter endotracheal tube was immediately and smoothly performed, the tip placed at a depth of 21 cm, and the tube positioned at the corner of the mouth, her tidal volume remained low at approximately 10 ml, despite high airway pressure (30 cmH_2_O). Furthermore, SpO_2_ decreased from 100 to 87% in approximately 5 min after tracheal intubation.

**Fig. 3:**
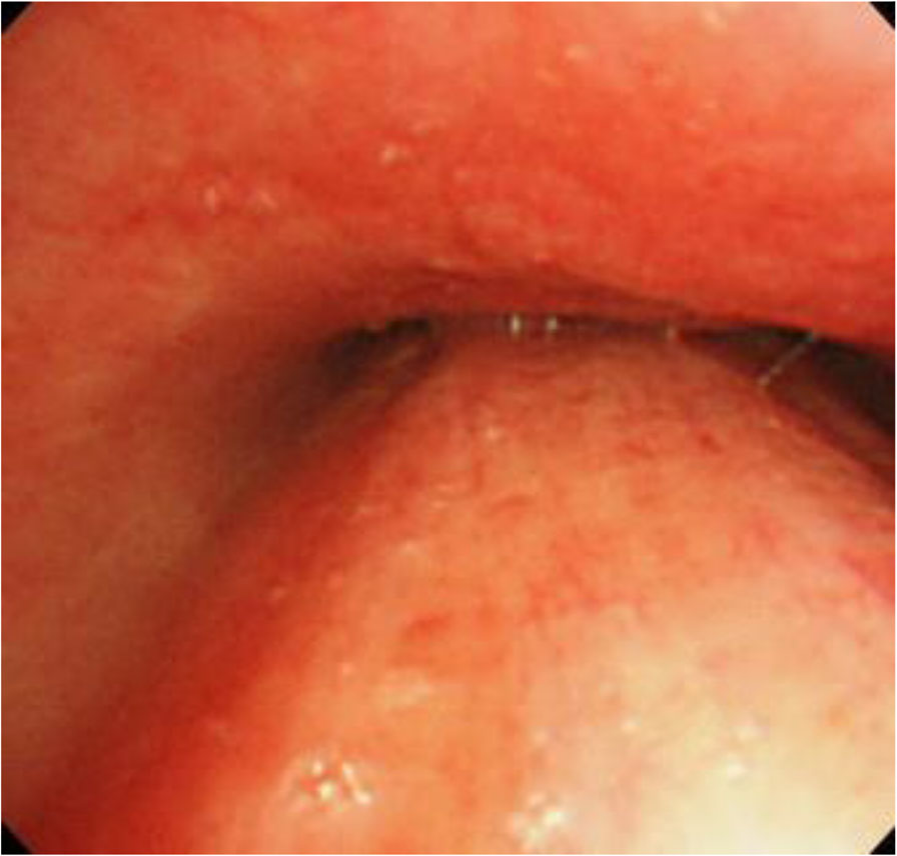
Bronchoscopic view of the lower trachea

A flexible bronchoscope (outer diameter of 4.9 mm) was inserted to confirm correct tracheal intubation, but the trachea was almost obstructed as it was being squeezed back and forth at the level of the carina (Fig. 3). While observing the area near the obstruction, we found that positive airway ventilation was indeed working and we hypothesized that the patency of the trachea had been improved when the bronchoscope had been inserted to a depth beyond the tumor, which then resulted in successful positive ventilation. A guidewire for the insertion of the balloon and stents was inserted through the internal lumen of the bronchoscope in a way the tip of the wire was positioned beyond the squeezed trachea as observed under X-ray fluoroscopy. Immediately after the removal of the bronchoscope, balloon dilation of the trachea was completed. Next, with the tracheal tube in place, stents were placed at the lower trachea and the left main bronchus under positive ventilation (tidal volume 230 with peak inspiratory pressure of 26 mmH_2_O). Her SpO_2_ remained at 100% during the procedure and the CO_2_ value was approximately 35 mmHg. FiO2 was maintained at 1.0 during anesthesia and after inserting the bronchoscope. V-V ECMO was terminated after the procedure, and her tidal volume increased to 300 mL at a peak inspiratory pressure of 13 mmH_2_O. Duration of the procedure and run-time for V-V ECMO were 70 and 132 min, respectively.

The patient was transferred to the intensive care unit where she remained sedated with intravenous dexmedetomidine and fentanyl until the next morning. After recovering from sedation, the patient’s trachea was extubated. Her respiratory status had improved dramatically, which permitted her to lie in a supine position without dyspnea. She underwent chemotherapy after the procedure, but the tumor continued to grow. Based on the results of the biopsy and tumor marker values, a diagnosis of extragonadal primary germ cell tumor was made. She developed disseminated intravascular coagulation and passed away on postoperative day 30.

## Discussion

When appropriate airway management and artificial ventilation are expected to be difficult following the induction of general anesthesia, the use of extracorporeal circulation before anesthesia induction can be an option. Usually, the flow rate of V-V ECMO is set 60–80 mL/kg/min [3] to maintain appropriate SpO_2_ values; however, in the current case, V-V ECMO flow rate was low and could not be improved even after cannula re-positioning, probably because of obstructed drainage secondary to compression by the tumor. Nonetheless, we expected that sufficient oxygenation would be possible and decided to induce anesthesia because, according to ASA Practice Guidelines for Management of the Difficult Airway [4], if ventilation is inadequate following the induction of anesthesia, consideration should be given to awakening the patient. Hence, we planned to awaken the patient and immediately resume spontaneous breathing if sufficient oxygenation could not be maintained because of alveolar hypoventilation or central airway collapse associated with the cessation of spontaneous breathing and positive pressure ventilation despite the presence of a secure airway.

Unexpectedly, positive pressure ventilation was not possible in our patient after the induction of anesthesia, and her hypoxemia progressed. We first performed tracheal intubation to rule out the possibility of difficult ventilation due to upper airway obstruction and to ensure a secure airway, and importantly, a bronchoscope was used to ensure that the tube had been positioned correctly. Although not initially intended, the insertion of the bronchoscope through the site of the obstruction improved tracheal patency and permitted positive pressure ventilation, allowing oxygen to be administered through the lungs. Thus, we were able to continue the planned procedure without awakening the patient. However, in a similar situation, if the bronchoscope is intentionally used to release a collapsed lumen, utmost care should be taken to avoid damage to the tracheal lumen. It was thought that the insertion of the bronchoscope not only relieved the physical pressure on the airway from the anterior-posterior direction, but also created a path for the air to flow on both sides of the bronchoscope. The ventilation status may have been further improved by inserting the tracheal tube beyond the narrowing portion because the cross-sectional area of the airway that contributes to ventilation increases. However, in this case, as the patient improved only with the insertion of the bronchoscope, we assumed that the planned treatment was possible and did not change the position of the tracheal tube. High-frequency jet ventilation (HFJV) is another strategy that can be used to increase oxygenation through the airway [5]. If inadequate ventilation persists, a catheter may be placed beyond the point of narrowing or obstruction and HFJV initiated; however, attention must be paid to prevent pressure trauma to the lungs.

## Conclusion

We describe a case where tracheal patency was transiently maintained by inserting a flexible bronchoscope in a patient with a trachea compressed by the presence of an anterior mediastinal mass. The patient showed decreased oxygenation after anesthesia induction despite V-V ECMO initiation.

## Data Availability

Not applicable

## Abbreviations

V-V ECMO: Veno-venous extracorporeal membrane oxygenation
HFJV: High-frequency jet ventilation
